# Attention mechanism-enhanced virtual reality scene generation for innovation and entrepreneurship education with cognitive load balancing

**DOI:** 10.1038/s41598-026-52481-z

**Published:** 2026-05-10

**Authors:** Xiaoxi Ge, Rongting Qin, Xiaojie Zi

**Affiliations:** College of Economy Management, Chongqing University of Arts and Sciences, Yongchuan District, Chongqing, 402160 China

**Keywords:** Virtual reality, Attention mechanism, Cognitive load balancing, Entrepreneurship education, Scene generation, Adaptive learning, Mathematics and computing, Psychology, Psychology

## Abstract

Virtual reality (VR) technology has emerged as a transformative tool for innovation and entrepreneurship education, yet existing systems face critical limitations in scene generation realism and cognitive load management. This research proposes an integrated framework combining attention mechanism-enhanced scene generation with adaptive cognitive load balancing strategies to address these challenges. The framework incorporates a multi-scale attention architecture operating across spatial, channel, and temporal dimensions to selectively emphasize pedagogically critical entrepreneurial elements while maintaining contextual coherence. A real-time cognitive load assessment system monitors physiological signals and behavioral patterns to implement dynamic scene complexity adjustments, maintaining learners within optimal challenge zones. Experimental validation across 45 entrepreneurship scenarios with 120 participants demonstrates substantial improvements: scene quality scores increased by 26–45% compared to baseline methods, error rates decreased by 60.9%, and task completion times reduced by 23.7%. The system maintained stable cognitive load within target ranges for 87.3% of session duration during extended 60-minute training sessions. Ablation studies confirm the essential contributions of individual attention components, with quality degradation of 12–18% upon removal. These findings advance both computational innovation in generative VR systems and pedagogical methodologies for entrepreneurship education, providing practical solutions for scalable, personalized training applications across diverse educational and professional development contexts.

## Introduction

Virtual reality (VR) technology has emerged as a transformative tool in innovation and entrepreneurship education, providing immersive experiential learning environments that enable students to simulate complex business scenarios without real-world financial risks^1^. The ability to generate realistic entrepreneurial scenarios in VR systems facilitates experiential learning by allowing learners to engage in decision-making processes, market analysis, and resource management within controlled virtual environments^2^. However, the effectiveness of VR-based entrepreneurship education critically depends on both the quality of scene generation and the management of user cognitive load during immersive experiences^3^.

### Research status and background

Recent advances in VR scene generation have primarily focused on geometric modeling and physics-based rendering techniques, yet these approaches often struggle to capture the dynamic complexity and contextual richness characteristic of real entrepreneurship environments^4^. Attention mechanisms, originally developed for natural language processing and computer vision tasks, have demonstrated remarkable capabilities in selectively focusing on salient features within complex data structures^5^. Several studies have explored the integration of attention mechanisms into generative models for improving visual content quality and semantic coherence^6^. However, the application of attention mechanisms specifically for entrepreneurship scenario generation in VR contexts remains largely unexplored, representing a significant research gap in both computational and educational domains^7^.

### Current challenges

Contemporary VR entrepreneurship training platforms face two critical limitations. First, automatically generated scenes frequently exhibit insufficient realism in representing authentic business environments, including inadequate modeling of stakeholder interactions, market dynamics, and contextual decision-making scenarios^8^. Second, users often experience excessive cognitive load due to information overload, complex interface navigation, and simultaneous processing demands across multiple sensory channels, which significantly impairs learning outcomes and user engagement^9^. These challenges necessitate innovative approaches that can simultaneously enhance scene generation quality while maintaining optimal cognitive load levels for effective learning experiences.

### Research significance and objectives

Addressing these challenges through attention mechanism-enhanced VR scene generation offers substantial theoretical and practical value. By enabling selective emphasis on pedagogically relevant entrepreneurial elements while suppressing irrelevant details, attention-based models can generate more contextually appropriate and educationally effective scenarios. Furthermore, dynamic cognitive load balancing strategies informed by attention mechanisms can adapt scene complexity and information presentation rates to individual user capabilities, thereby optimizing learning efficiency. This research contributes to both computational innovation in generative VR systems and pedagogical advancement in entrepreneurship education methodologies^10^.

This paper proposes an attention mechanism-enhanced framework for VR entrepreneurship scene generation integrated with adaptive cognitive load balancing strategies. The primary innovations include: (1) a multi-head attention architecture specifically designed for generating contextually rich entrepreneurship scenarios, (2) a cognitive load assessment model based on physiological signals and interaction patterns, and (3) a dynamic scene adaptation mechanism that maintains optimal learning conditions through real-time cognitive load regulation. These contributions advance the state-of-the-art in intelligent VR education systems while providing practical solutions for scalable entrepreneurship training applications.

## Related theory and technical foundation

### Virtual reality scene generation technology

Virtual reality scene generation encompasses the computational processes of creating three-dimensional environments through geometric modeling, texture synthesis, and spatial layout optimization^11^. The fundamental pipeline typically involves scene representation, content generation, and real-time rendering, where each component requires careful balance between visual fidelity and computational efficiency^12^.

Rule-based scene generation approaches rely on predefined heuristics and domain-specific constraints to construct virtual environments through deterministic algorithms. These methods employ L-systems for vegetation modeling, shape grammars for architectural structures, and constraint satisfaction mechanisms for spatial arrangement^13^. While rule-based techniques ensure consistency and controllability, they often lack the diversity and naturalism required for complex entrepreneurial scenarios. Conversely, data-driven methods leverage large-scale datasets to learn statistical patterns and generate scenes through machine learning models^14^. These approaches can capture nuanced environmental characteristics but require substantial training data and computational resources.

Procedural generation technology has become instrumental in VR scene construction by enabling algorithmic creation of large-scale environments with minimal manual intervention. The procedural content generation process can be formalized as a mapping function:

1 $$\:S=f(P,R)$$

where $\:S$ represents the generated scene, $\:P$ denotes the parameter set controlling generation characteristics, and $\:R$ represents random seeds ensuring variability^15^. This technique significantly reduces content creation costs while maintaining acceptable quality levels for interactive applications.

Generative Adversarial Networks (GANs) have demonstrated superior performance in scene generation by learning complex data distributions through adversarial training processes. The GAN framework optimizes a minimax objective function (well-known formula):

2 $$\:{\mathrm{m}\mathrm{i}\mathrm{n}}_{G}{\mathrm{m}\mathrm{a}\mathrm{x}}_{D}V(D,G)={\mathbb{E}}_{x\sim{p}_{data}\left(x\right)}\left[\mathrm{l}\mathrm{o}\mathrm{g}D\right(x\left)\right]+{\mathbb{E}}_{z\sim{p}_{z}\left(z\right)}\left[\mathrm{l}\mathrm{o}\mathrm{g}\right(1-D\left(G\right(z\left)\right)\left)\right]$$

where $\:G$ represents the generator network, $\:D$ denotes the discriminator, and $\:z$ signifies the latent input vector^16^. GANs excel at generating high-resolution textures and realistic spatial layouts, making them particularly suitable for immersive VR applications.

Innovation and entrepreneurship scenarios impose unique generation requirements including dynamic stakeholder interactions, temporally evolving market conditions, and context-sensitive decision points. These scenarios must simultaneously represent physical business environments, abstract data visualizations, and interpersonal communication contexts^17^. The generation difficulty stems from balancing semantic coherence across multiple representation modalities while maintaining computational efficiency for real-time interaction. Traditional generation methods struggle to capture the multifaceted nature of entrepreneurial activities, necessitating specialized approaches that integrate domain knowledge with advanced generative techniques.

### Attention mechanism theory and applications

Attention mechanisms originate from biological visual processing systems where organisms selectively concentrate on salient information while filtering irrelevant stimuli to optimize cognitive resource allocation^18^. Computational attention models mathematically simulate this selective processing by assigning differential weights to input features based on their relevance to specific tasks^19^.

Self-attention mechanisms enable models to capture dependencies within a single sequence by computing relationships among all elements regardless of their positional distances. The fundamental self-attention operation computes attention weights through the scaled dot-product formulation (well-known formula):

3 $$\:\mathrm{A}\mathrm{t}\mathrm{t}\mathrm{e}\mathrm{n}\mathrm{t}\mathrm{i}\mathrm{o}\mathrm{n}(Q,K,V)=\mathrm{s}\mathrm{o}\mathrm{f}\mathrm{t}\mathrm{m}\mathrm{a}\mathrm{x}\left(\frac{Q{K}^{T}}{\sqrt[]{{d}_{k}}}\right)V$$

where $\:Q$, $\:K$, and $\:V$ represent query, key, and value matrices respectively, and $\:{d}_{k}$ denotes the dimensionality of key vectors^5^. This mechanism allows each position to attend to all positions in the input, effectively capturing long-range dependencies crucial for understanding complex spatial or semantic relationships.

Multi-head attention extends self-attention by performing parallel attention operations with different learned projections, enabling the model to jointly attend to information from different representation subspaces. The multi-head attention output is computed as (well-known formula):

4 $$\:\mathrm{M}\mathrm{u}\mathrm{l}\mathrm{t}\mathrm{i}\mathrm{H}\mathrm{e}\mathrm{a}\mathrm{d}(Q,K,V)=\mathrm{C}\mathrm{o}\mathrm{n}\mathrm{c}\mathrm{a}\mathrm{t}({\mathrm{h}\mathrm{e}\mathrm{a}\mathrm{d}}_{1},...,{\mathrm{h}\mathrm{e}\mathrm{a}\mathrm{d}}_{h}){W}^{O}$$

where $\:{\mathrm{h}\mathrm{e}\mathrm{a}\mathrm{d}}_{i}=\mathrm{A}\mathrm{t}\mathrm{t}\mathrm{e}\mathrm{n}\mathrm{t}\mathrm{i}\mathrm{o}\mathrm{n}(Q{W}_{i}^{Q},K{W}_{i}^{K},V{W}_{i}^{V})$ and $\:{W}^{O}$ represents the output projection matrix^5^. Cross-attention mechanisms facilitate interaction between different modalities or sequences by using queries from one sequence and keys-values from another, proving particularly effective for multi-modal fusion tasks.

Attention mechanisms have achieved breakthrough performance in computer vision tasks including object detection, image segmentation, and visual question answering by enabling models to focus on discriminative regions within images^20^. In natural language processing, attention-based architectures have revolutionized machine translation, text summarization, and language understanding by capturing contextual dependencies more effectively than recurrent approaches^21^.

Attention weight calculation methods employ various similarity metrics including dot product, additive scoring, and bilinear transformations to quantify relevance between query and key vectors. Feature enhancement strategies leverage computed attention weights to amplify salient features while suppressing background noise through element-wise multiplication or gated mechanisms. For scene generation applications, attention mechanisms offer substantial potential by identifying critical scene elements that define entrepreneurial contexts, prioritizing generation of semantically coherent business environments, and dynamically adjusting content complexity based on user attention patterns^22^. These capabilities align directly with requirements for generating contextually rich and educationally effective VR entrepreneurship scenarios.

### Cognitive load theory and measurement methods

Cognitive Load Theory (CLT) posits that human working memory possesses limited capacity for processing information, and effective learning occurs when instructional designs optimize the allocation of cognitive resources^23^. The theory delineates three distinct types of cognitive load: intrinsic cognitive load, determined by the inherent complexity of learning materials and the learner’s prior knowledge; extraneous cognitive load, imposed by suboptimal presentation formats and instructional designs; and germane cognitive load, dedicated to schema construction and automation processes that facilitate meaningful learning^24^.

Virtual reality environments introduce unique cognitive load factors including spatial navigation demands, multisensory information processing requirements, stereoscopic depth perception challenges, and real-time interaction complexities. These factors collectively intensify the overall cognitive burden experienced by users, particularly when VR scenarios incorporate dense informational elements or require simultaneous attention to multiple task dimensions^25^. The total cognitive load in VR contexts can be conceptualized as:

5 $$\:C{L}_{total}=C{L}_{intrinsic}+C{L}_{extraneous}+C{L}_{germane}$$

where optimal learning efficiency requires that $\:C{L}_{total}\le\:{C}_{capacity}$, with $\:{C}_{capacity}$ representing individual working memory capacity^26^.

Cognitive load assessment employs three complementary methodological approaches. Subjective measurement methods utilize self-report instruments such as the NASA Task Load Index (NASA-TLX) and Paas scale, where participants rate perceived mental effort on Likert-type scales. Objective measurement techniques analyze behavioral indicators including task performance metrics, response times, error rates, and secondary task performance to infer cognitive resource allocation. Physiological measurement approaches monitor bio-signals including pupil dilation, heart rate variability, electroencephalography (EEG) patterns, and galvanic skin response to quantify cognitive workload^27^. The effectiveness of each method varies depending on measurement context, with physiological indicators offering continuous real-time assessment advantages for VR applications.

In VR entrepreneurship scenarios, excessive cognitive load manifests through decreased decision-making quality, prolonged task completion times, increased error frequencies, and premature termination of learning sessions. When total cognitive load exceeds working memory capacity, learners experience cognitive overload, severely impairing information encoding, knowledge integration, and skill acquisition processes^28^. The relationship between cognitive load and learning performance follows an inverted U-shaped function:

6 $$\:P=\alpha\: \cdot CL-\beta\: \cdot C{L}^{2}$$

where $\:P$ represents learning performance, and parameters $\:\alpha\:$ and $\:\beta\:$ determine the optimal cognitive load level. Maintaining cognitive load balance is essential for sustaining user immersion, as excessive load triggers discomfort and disengagement while insufficient load results in boredom and reduced motivation. Effective cognitive load management ensures learners remain within the optimal challenge zone, maximizing both educational outcomes and experiential quality in VR-based entrepreneurship training.

## Attention mechanism-enhanced VR entrepreneurship scene generation model

### Overall framework design and scene generation process

The proposed attention mechanism-enhanced VR entrepreneurship scene generation framework integrates four core functional modules to achieve context-aware, adaptive content creation while maintaining optimal cognitive load levels^29^. This framework employs a hierarchical architecture that progressively transforms abstract user requirements into concrete, immersive virtual environments through attention-guided feature selection and synthesis processes.

The scene requirement analysis module serves as the initial processing layer, parsing user inputs including entrepreneurial domain specifications, difficulty levels, target learning objectives, and contextual constraints. This module employs natural language understanding techniques to extract semantic features from textual requirements and maps them onto structured scene parameters through a transformation function:

7 $$\:r=\phi\:(u,k)$$

where $\:r$ represents the structured requirement vector, $\:u$ denotes raw user input, $\:k$ signifies domain knowledge embeddings, and $\:\phi\:$ represents the parsing function^30^.

The scene element extraction module identifies and retrieves relevant scene components from a comprehensive entrepreneurship element database including physical objects, spatial layouts, stakeholder avatars, and interactive event sequences. This module implements content-based retrieval augmented with semantic matching to ensure contextual appropriateness of selected elements. The attention enhancement generation module constitutes the core innovation of this framework, utilizing multi-head attention mechanisms to selectively emphasize pedagogically significant scene features while suppressing irrelevant details. The attention-weighted generation process computes scene feature representations as:

8 $$\:{f}_{scene}=\sum\:_{i=1}^{N}{\alpha\:}_{i} \cdot {e}_{i}$$

where $\:{e}_{i}$ represents the $\:i$-th scene element embedding, $\:{\alpha\:}_{i}$ denotes the computed attention weight, and $\:N$ indicates the total number of elements^31^. The cognitive load assessment module continuously monitors user physiological signals and behavioral indicators, computing real-time cognitive load metrics to inform adaptive scene complexity adjustments.

Table 1 summarizes the functional specifications and data interfaces for each module within the generation framework. As presented in Table 1, the modular architecture ensures clear separation of concerns while enabling seamless data flow through well-defined interfaces.

**Table 1:** Scene Generation Module Functions and Data Interfaces.

Module Name	Primary Function	Input Data	Output Data
Scene Requirement Analysis	Parse user requirements and extract semantic features	User input text, domain ontologies	Structured requirement vectors
Scene Element Extraction	Retrieve and match relevant scene components	Requirement vectors, element database	Candidate element sets
Attention Enhancement Generation	Synthesize scenes with attention-guided feature weighting	Element sets, attention parameters	Generated VR scenes
Cognitive Load Assessment	Monitor and evaluate user cognitive workload	Physiological signals, interaction logs	Cognitive load metrics

**Fig. 1:**
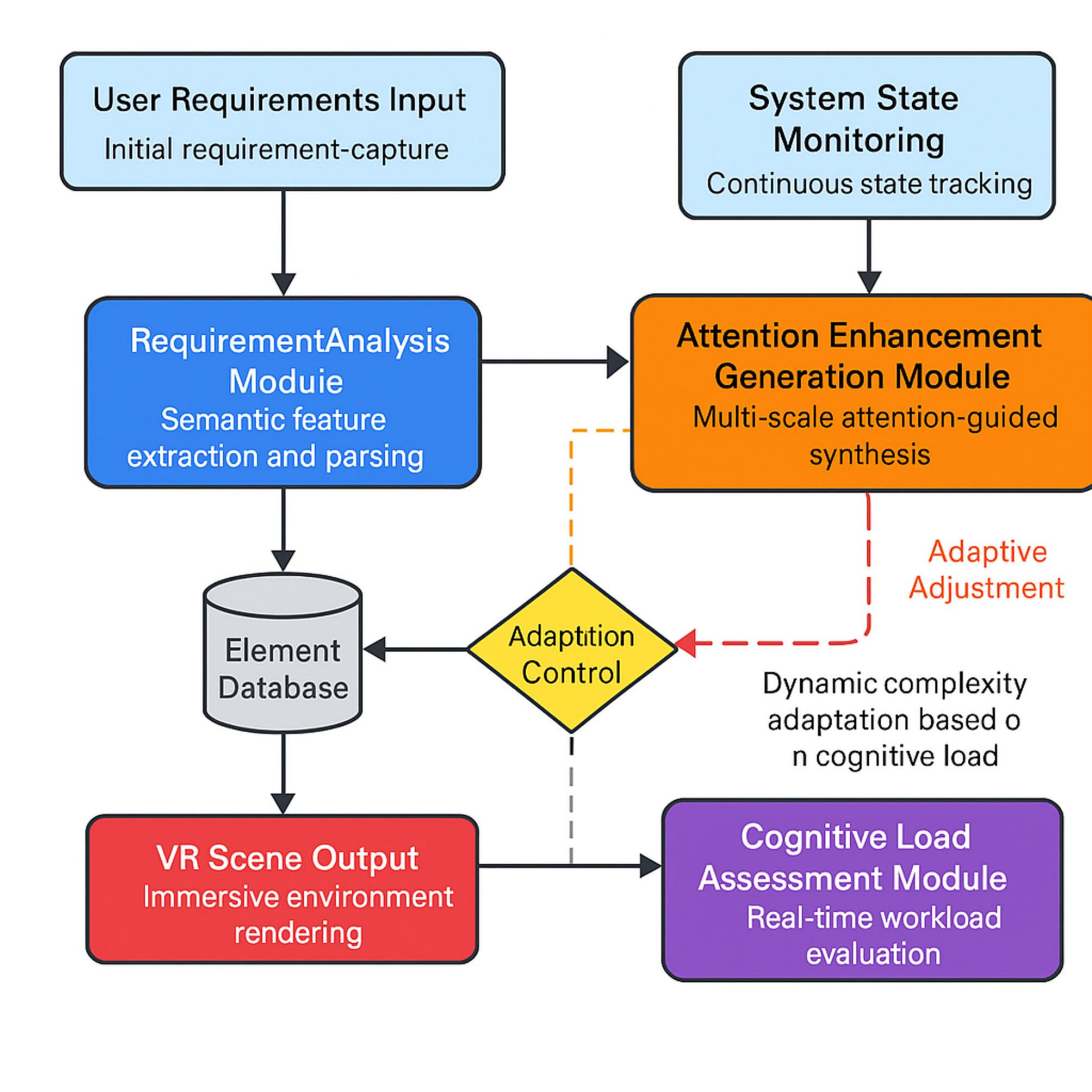
Attention Mechanism-Enhanced VR Scene Generation Framework and Process Flow.

Figure 1 illustrates the complete scene generation pipeline from initial requirement input to final VR environment deployment. As shown in Fig. 1, the generation process follows a feed-forward architecture with feedback loops enabling iterative refinement based on cognitive load assessments.

The multi-level architecture comprises three operational layers: the perception layer captures user inputs and system states; the processing layer executes attention-based feature extraction and scene synthesis; and the presentation layer renders generated content while monitoring cognitive responses^32^. Key technical nodes include the attention weight computation module, feature fusion mechanism, and adaptive complexity controller. Data flows bidirectionally between the cognitive load assessment module and the generation module, enabling dynamic scene adaptation through a feedback control mechanism expressed as:

9 $$\:{s}_{t+1}={s}_{t}+\lambda\: \cdot {\nabla\:}_{CL}\left({s}_{t}\right)$$

where $\:{s}_{t}$ represents scene parameters at time $\:t$, $\:\lambda\:$ denotes the adaptation rate, and $\:{\nabla\:}_{CL}$ indicates the gradient with respect to cognitive load deviation from optimal levels^33^. This closed-loop control ensures continuous alignment between scene complexity and user cognitive capacity throughout the learning session.

### Multi-scale attention enhancement mechanism

The multi-scale attention enhancement mechanism employs three complementary attention modules operating across spatial, channel, and temporal dimensions to comprehensively capture salient features within entrepreneurship scene representations^34^. This hierarchical attention architecture enables the model to selectively emphasize contextually relevant scene elements while maintaining computational efficiency for real-time VR applications.

The spatial attention module identifies critical regions within scene layouts by computing location-specific importance weights across the spatial domain. Given an input feature map $\:F\in\:{\mathbb{R}}^{H\times\:W\times\:C}$ where $\:H$, $\:W$, and $\:C$ denote height, width, and channel dimensions respectively, the spatial attention map $\:{M}_{s}\in\:{\mathbb{R}}^{H\times\:W}$ is computed as:

10 $$\:{M}_{s}=\sigma\:\left({f}^{7\times\:7}\right(\left[\mathrm{A}\mathrm{v}\mathrm{g}\mathrm{P}\mathrm{o}\mathrm{o}\mathrm{l}\right(F);\mathrm{M}\mathrm{a}\mathrm{x}\mathrm{P}\mathrm{o}\mathrm{o}\mathrm{l}(F\left)\right]\left)\right)$$

where $\:\sigma\:$ represents the sigmoid activation function, $\:{f}^{7\times\:7}$ denotes a convolutional layer with $\:7\times\:7$ kernel, and the pooling operations aggregate channel-wise statistics^34^. This mechanism effectively highlights regions containing critical entrepreneurial elements such as customer interaction zones, product display areas, and decision-making interfaces.

The channel attention module performs adaptive feature selection by evaluating the relative importance of different feature channels, each representing distinct semantic attributes of scene elements. The channel attention weights $\:{M}_{c}\in\:{\mathbb{R}}^{C}$ are derived through a squeeze-and-excitation operation:

11 $$\:{M}_{c}=\sigma\:({W}_{2} \cdot \mathrm{R}\mathrm{e}\mathrm{L}\mathrm{U}({W}_{1} \cdot \mathrm{G}\mathrm{A}\mathrm{P}\left(F\right)\left)\right)$$

where $\:\mathrm{G}\mathrm{A}\mathrm{P}$ denotes global average pooling, $\:{W}_{1}\in\:{\mathbb{R}}^{\frac{C}{r}\times\:C}$ and $\:{W}_{2}\in\:{\mathbb{R}}^{C\times\:\frac{C}{r}}$ represent dimension reduction and expansion matrices with reduction ratio $\:r$, and $\:\mathrm{R}\mathrm{e}\mathrm{L}\mathrm{U}$ provides non-linear transformation^35^. Channel attention enables the model to prioritize features corresponding to pedagogically significant scene attributes including stakeholder behaviors, market indicators, and resource constraints.

The temporal attention mechanism processes sequential information inherent in dynamic entrepreneurship scenarios where business conditions evolve across multiple time steps. For a temporal feature sequence $\:\{{F}_{1},{F}_{2},...,{F}_{T}\}$, temporal attention weights $\:{\boldsymbol{\alpha\:}}_{t}$ are computed using a self-attention framework:

12 $$\:{\boldsymbol{\alpha\:}}_{t}=\mathrm{s}\mathrm{o}\mathrm{f}\mathrm{t}\mathrm{m}\mathrm{a}\mathrm{x}\left(\frac{{Q}_{t}{K}^{T}}{\sqrt[]{{d}_{k}}}\right)$$

where $\:{Q}_{t}$ and $\:K$ represent query and key projections of temporal features, enabling the model to capture dependencies between current scene states and historical contexts^36^. This temporal modeling capability ensures continuity in generated scenarios where entrepreneurial decisions have consequences that propagate through time.

Table 2 presents the configuration parameters for each attention module within the multi-scale framework. As presented in Table 2, the parameter settings balance computational complexity against feature extraction capability, with spatial attention employing larger receptive fields for contextual understanding while channel attention utilizes dimensionality reduction for efficiency.

**Table 2:** Attention Module Configuration Parameters.

Attention Module	Input Dimension	Kernel Size/Layers	Reduction Ratio	Output Dimension
Spatial Attention	$\:H\times\:W\times\:C$	$\:7\times\:7$	N/A	$\:H\times\:W\times\:1$
Channel Attention	$\:H\times\:W\times\:C$	FC layers: $\:C\to\:\frac{C}{16}\to\:C$	16	$\:1\times\:1\times\:C$
Temporal Attention	$\:T\times\:{d}_{model}$	Multi-head: 8 heads	N/A	$\:T\times\:{d}_{model}$

**Fig. 2:**
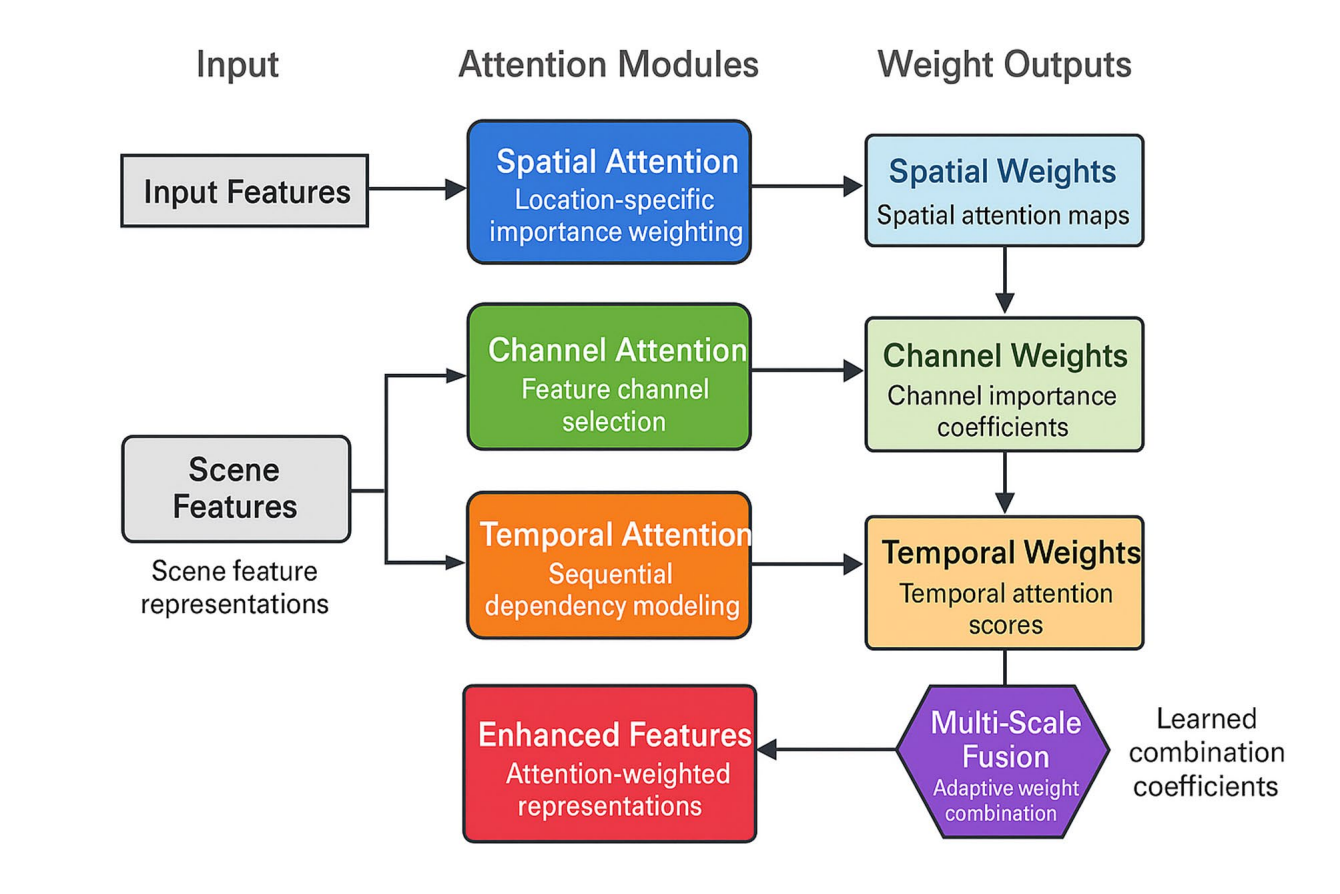
Multi-Scale Attention Mechanism Architecture and Feature Fusion Process.

Figure 2 illustrates the architectural organization of the multi-scale attention mechanism and its integration pathway within the scene generation pipeline. As shown in Fig. 2, the three attention modules operate in parallel on input features, with their outputs subsequently fused through an adaptive weighting strategy that determines the relative contribution of each attention type based on scene characteristics.

The multi-scale feature fusion strategy aggregates attention outputs through learned combination coefficients that adapt to different scene generation contexts. The fused attention-enhanced features are obtained by element-wise multiplication of original features with the composite attention map, effectively amplifying salient information while suppressing irrelevant variations. This attention-guided enhancement significantly improves the realism of generated entrepreneurship scenes by ensuring that critical business elements such as customer demographics, competitive landscapes, and resource availability receive appropriate representational emphasis^37^. The rationality of scene configurations is enhanced through channel attention’s selective activation of semantically coherent feature combinations, preventing the generation of implausible business scenarios such as conflicting market conditions or inconsistent stakeholder behaviors. The integrated multi-scale attention framework thus establishes a robust mechanism for producing contextually appropriate, educationally effective VR entrepreneurship environments.

### Cognitive load balancing strategy

The cognitive load balancing strategy integrates predictive modeling, dynamic adjustment mechanisms, and real-time feedback control to maintain user cognitive workload within optimal operational zones throughout VR entrepreneurship training sessions^38^. This comprehensive approach addresses the fundamental tension between providing informationally rich learning environments and preventing cognitive overload that impairs knowledge acquisition.

The user feature-based cognitive load prediction model estimates individual cognitive capacity by analyzing prior performance metrics, domain expertise levels, working memory capacity assessments, and demographic characteristics. The prediction function employs a supervised learning framework trained on historical user interaction data:

13 $$\\hat :{CL}\left(t\right)=g({u}_{feat},{s}_{param},{h}_{t-1})$$

where $$\\hat :{CL}\left(t\right)$$ represents predicted cognitive load at time $\:t$, $\:{u}_{feat}$ denotes the user feature vector, $\:{s}_{param}$ indicates current scene parameters, $\:{h}_{t-1}$ captures historical interaction patterns, and $\:g$ represents the prediction function implemented through neural networks^39^. This predictive capability enables proactive scene adjustments before cognitive overload manifests in degraded performance.

The scene complexity dynamic adjustment algorithm modulates multiple scene attributes including visual detail density, information channel multiplicity, interaction task concurrency, and decision-making time constraints. Complexity adjustments follow a gradient descent optimization that minimizes deviation from target cognitive load while preserving educational content integrity. The information presentation density adaptive control strategy regulates the rate and volume of information displayed to users through temporal gating mechanisms that selectively reveal scene elements based on attention focus and task progression. This prevents information flooding while ensuring critical entrepreneurial data remains accessible when needed.

The interaction task difficulty grading mechanism categorizes entrepreneurial activities into hierarchical complexity levels spanning from basic operational tasks to complex strategic decision-making challenges. Task assignment follows a dynamic difficulty adjustment protocol that increases challenge levels as user proficiency improves, implementing zone of proximal development principles to optimize learning efficiency^40^. Each difficulty grade specifies permissible ranges for task parameters including time pressure, information ambiguity, stakeholder conflict intensity, and consequence severity.

Table 3 summarizes the multi-dimensional cognitive load assessment indicator system employed for real-time monitoring. Table 3 shows that the assessment framework integrates subjective, behavioral, and physiological metrics to provide comprehensive cognitive state evaluation across multiple measurement domains.

**Table 3:** Cognitive Load Assessment Indicator System.

Indicator Category	Measurement Metric	Assessment Method
Subjective Load	Perceived mental effort	NASA-TLX scale (0–100)
Task Performance	Completion time and accuracy	Behavioral log analysis
Interaction Patterns	Navigation efficiency and error rate	Interaction trace mining
Physiological Signals	Heart rate variability	ECG sensor monitoring
Visual Attention	Pupil diameter variation	Eye-tracking measurement
Neural Activity	EEG theta/alpha ratio	Electroencephalography

The real-time monitoring and feedback adjustment system continuously samples these indicators at regular intervals, computing aggregate cognitive load scores that trigger adaptive responses when thresholds are exceeded. The feedback controller implements proportional-integral-derivative control logic to achieve smooth, stable regulation without oscillatory behavior that could disrupt user experience^41^.

The cognitive load balance optimization objective function formalizes the dual requirements of maintaining appropriate challenge levels while preventing overload:

14 $$\:{\mathrm{m}\mathrm{i}\mathrm{n}}_{s}\mathcal{L}={\omega\:}_{1}(CL-C{L}_{target}{)}^{2}+{\omega\:}_{2} \cdot \mathcal{R}(s)+{\omega\:}_{3} \cdot \mathcal{C}(s)$$

where $\:CL$ represents measured cognitive load, $\:C{L}_{target}$ denotes the optimal load level, $\:\mathcal{R}\left(s\right)$ quantifies scene richness to prevent oversimplification, $\:\mathcal{C}\left(s\right)$ measures content coverage ensuring educational objectives are met, and $\:{\omega\:}_{i}$ are weighting coefficients balancing competing objectives. This formulation explicitly encodes the requirement to preserve scene richness while managing cognitive demands, preventing the system from trivially reducing load by eliminating educational content.

The progressive scene display scheme implements staged revelation of scene complexity following a scaffolded learning trajectory. Initial exposure presents simplified scenarios with limited decision variables and clear causal relationships, gradually introducing additional complexity dimensions as users demonstrate mastery of fundamental concepts^42^. This approach balances the learning curve by ensuring that intrinsic cognitive load from new material aligns with learner capacity development, while systematically reducing extraneous load through optimized information architecture and intuitive interface designs. The progressive strategy employs curriculum learning principles where scene difficulty sequences are automatically generated based on concept dependency graphs and individual learner progression rates, creating personalized learning paths that maximize knowledge retention while minimizing cognitive strain.

## Experimental validation and application analysis

### Experimental design and data collection

The experimental validation employed a comprehensive comparative study design encompassing three primary experimental conditions: baseline methods representing conventional VR scene generation approaches, the proposed attention-enhanced framework with full cognitive load balancing capabilities, and ablation experiments systematically removing individual components to assess their contributions. The baseline methods included rule-based procedural generation and standard GAN-based scene synthesis without attention mechanisms or cognitive load adaptation^43^. Ablation variants examined scenarios with attention mechanisms only (no cognitive balancing), cognitive balancing only (no attention enhancement), and individual attention module removals (spatial-only, channel-only, temporal-only configurations).

The test scenario repository comprised 45 distinct entrepreneurship contexts distributed across three primary domains to ensure comprehensive coverage of diverse business situations. The restaurant entrepreneurship domain included scenarios such as fast-food chain expansion, fine dining establishment management, and food delivery platform operations, each presenting unique challenges in customer service, inventory management, and competitive positioning. The technology entrepreneurship domain encompassed software startup development, hardware product launch scenarios, and platform ecosystem construction challenges, emphasizing innovation management, technical decision-making, and market entry strategies. The cultural entrepreneurship domain featured museum operation management, art gallery curation scenarios, and cultural event organization contexts, highlighting stakeholder engagement, resource constraints, and social impact considerations. Each scenario incorporated multiple difficulty levels ranging from introductory exercises to advanced strategic challenges, enabling assessment across varied complexity gradients.

The experimental environment utilized high-performance computing infrastructure to support real-time scene generation and rendering. Hardware configuration consisted of workstations equipped with NVIDIA RTX 4090 GPUs (24GB VRAM), Intel Core i9-13900 K processors (24 cores, 3.0 GHz base frequency), and 64GB DDR5 RAM to ensure computational capacity for attention mechanism calculations and physiological signal processing. VR display employed Meta Quest Pro headsets featuring 1800 × 1920 resolution per eye, 90 Hz refresh rate, 106-degree horizontal field of view, and integrated eye-tracking capabilities essential for gaze-based attention monitoring. The software platform integrated Unity 3D (version 2022.3 LTS) for scene rendering, PyTorch (version 2.0) for attention mechanism implementation, and custom middleware for cognitive load assessment and adaptive control^44^.

Participant recruitment followed stringent selection criteria to ensure sample validity and experimental reliability. Inclusion criteria specified participants aged 18–35 years with normal or corrected-to-normal vision, no history of motion sickness or VR intolerance, and basic entrepreneurship knowledge acquired through coursework or practical experience. A total of 120 participants (68 male, 52 female) were recruited through university announcement systems and professional networks, with demographic distribution spanning undergraduate students (*n* = 45), graduate students (*n* = 38), early-career entrepreneurs (*n* = 22), and business professionals (*n* = 15). Participants were randomly assigned to experimental conditions using stratified sampling to balance demographic characteristics across groups.

Table 4 presents the comprehensive experimental parameter configuration employed throughout the validation study. As presented in Table 4, parameters were calibrated through pilot testing to optimize system performance while maintaining realistic experimental conditions.

The scene generation quality evaluation framework integrated multiple objective and subjective metrics. Objective quality metrics included scene realism scores computed through Fréchet Inception Distance (FID) comparing generated scenes against real entrepreneurship environment photographs, semantic consistency measurements evaluating logical coherence of scene element combinations, and diversity indices quantifying variation across generated scenarios. The quality score $\:Q$ was computed as a weighted combination:

**Table 4:** Experimental Parameter Settings.

Parameter Category	Parameter Name	Value/Range	Unit
Scene Generation	Attention heads	8	-
Scene Generation	Feature dimension	512	Dimensions
Scene Generation	Generation resolution	2048 × 2048	Pixels
Cognitive Assessment	Sampling frequency	10	Hz
Cognitive Assessment	Target cognitive load	0.6–0.75	Normalized
Experimental Protocol	Session duration	20–30	Minutes
Experimental Protocol	Rest interval	5	Minutes

15 $$\:Q={\beta\:}_{1} \cdot {Q}_{realism}+{\beta\:}_{2} \cdot {Q}_{consistency}+{\beta\:}_{3} \cdot {Q}_{diversity}$$

where $\:{Q}_{realism}$, $\:{Q}_{consistency}$, and $\:{Q}_{diversity}$ represent normalized scores for each dimension, and $\:{\beta\:}_{i}$ denote weighting coefficients satisfying $\:\sum\:_{i=1}^{3}{\beta\:}_{i}=1$.

Cognitive load measurement employed a multi-modal data collection protocol integrating physiological monitoring, behavioral analysis, and subjective assessment. Physiological signals including electrocardiography (ECG), electroencephalography (EEG), and eye-tracking data were recorded continuously throughout experimental sessions using portable Shimmer3 ECG sensors, Emotiv EPOC + EEG headsets, and Quest Pro integrated eye trackers respectively. Behavioral metrics extracted from interaction logs encompassed task completion times, navigation path efficiency, error frequencies, and decision response latencies. The aggregate cognitive load index $\:C{L}_{agg}$ synthesized multiple indicators through principal component analysis:

16 $$\:C{L}_{agg}=\sum\:_{i=1}^{n}{w}_{i} \cdot \frac{{I}_{i}-{\mu\:}_{i}}{{\sigma\:}_{i}}$$

where $\:{I}_{i}$ represents the $\:i$-th raw indicator, $\:{\mu\:}_{i}$ and $\:{\sigma\:}_{i}$ denote population mean and standard deviation for normalization, $\:{w}_{i}$ indicates the principal component loading, and $\:n$ specifies the total number of indicators.

Subjective evaluation employed standardized questionnaires administered immediately following experimental sessions. The NASA Task Load Index (NASA-TLX) assessed perceived workload across six dimensions: mental demand, physical demand, temporal demand, performance, effort, and frustration. Scene quality perception was evaluated through custom Likert-scale instruments measuring realism, engagement, educational value, and contextual appropriateness on 7-point scales. The System Usability Scale (SUS) quantified overall user experience and interface effectiveness. Data collection procedures required participants to complete baseline assessments before VR exposure, undergo training sessions familiarizing them with interaction mechanisms, experience three randomly-ordered scenario types with counterbalanced condition assignments, and provide post-session evaluations for each condition. This rigorous protocol enabled robust statistical comparison across experimental conditions while controlling for learning effects and individual differences.

**Fig. 3:**
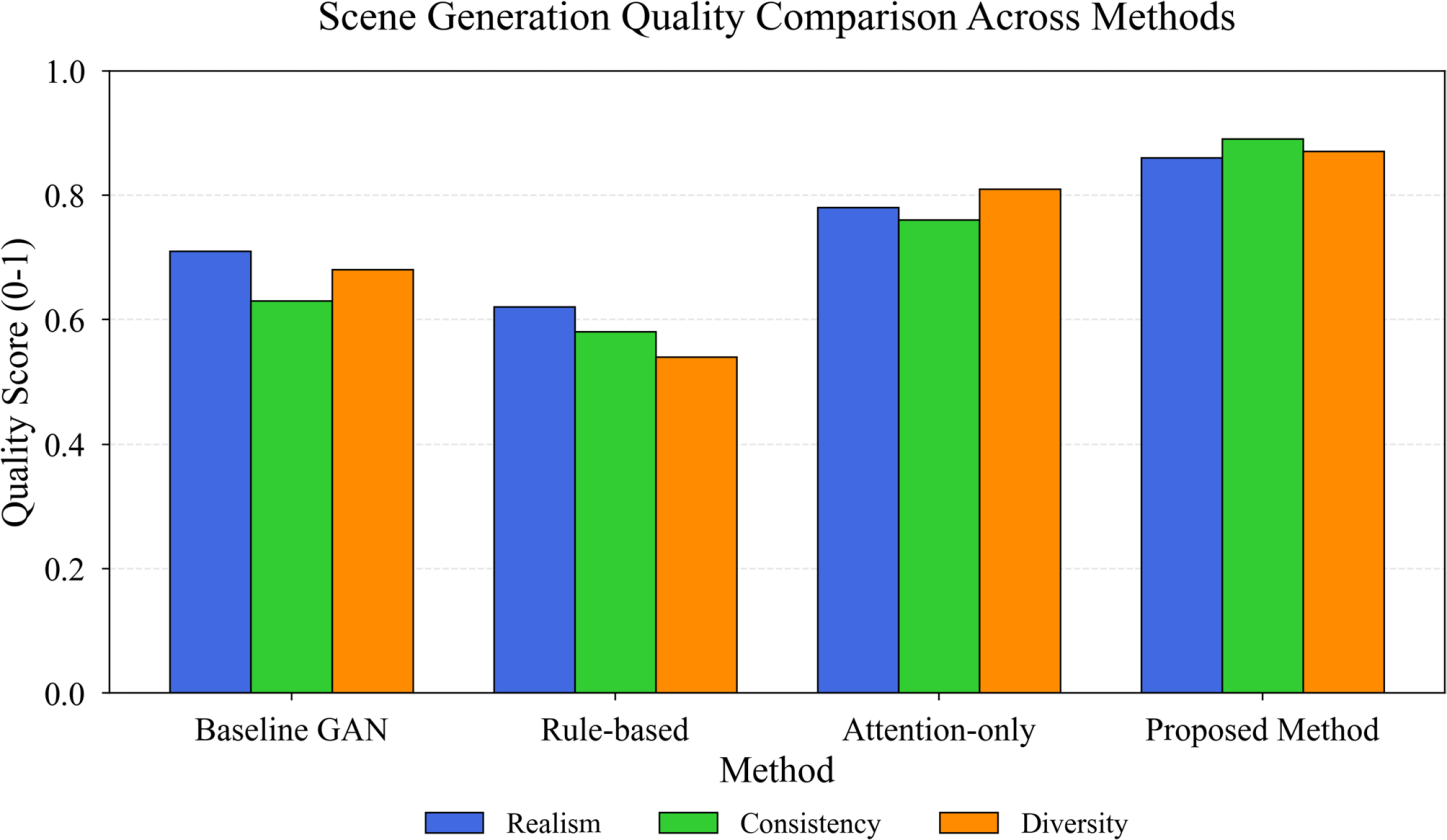
Scene Generation Quality Score Comparison Across Methods.

Figure 3 illustrates the comparative distribution of scene generation quality scores across experimental conditions. As shown in Fig. 3, the proposed attention-enhanced method consistently achieves superior performance across all evaluation metrics compared to baseline approaches.

**Fig. 4:**
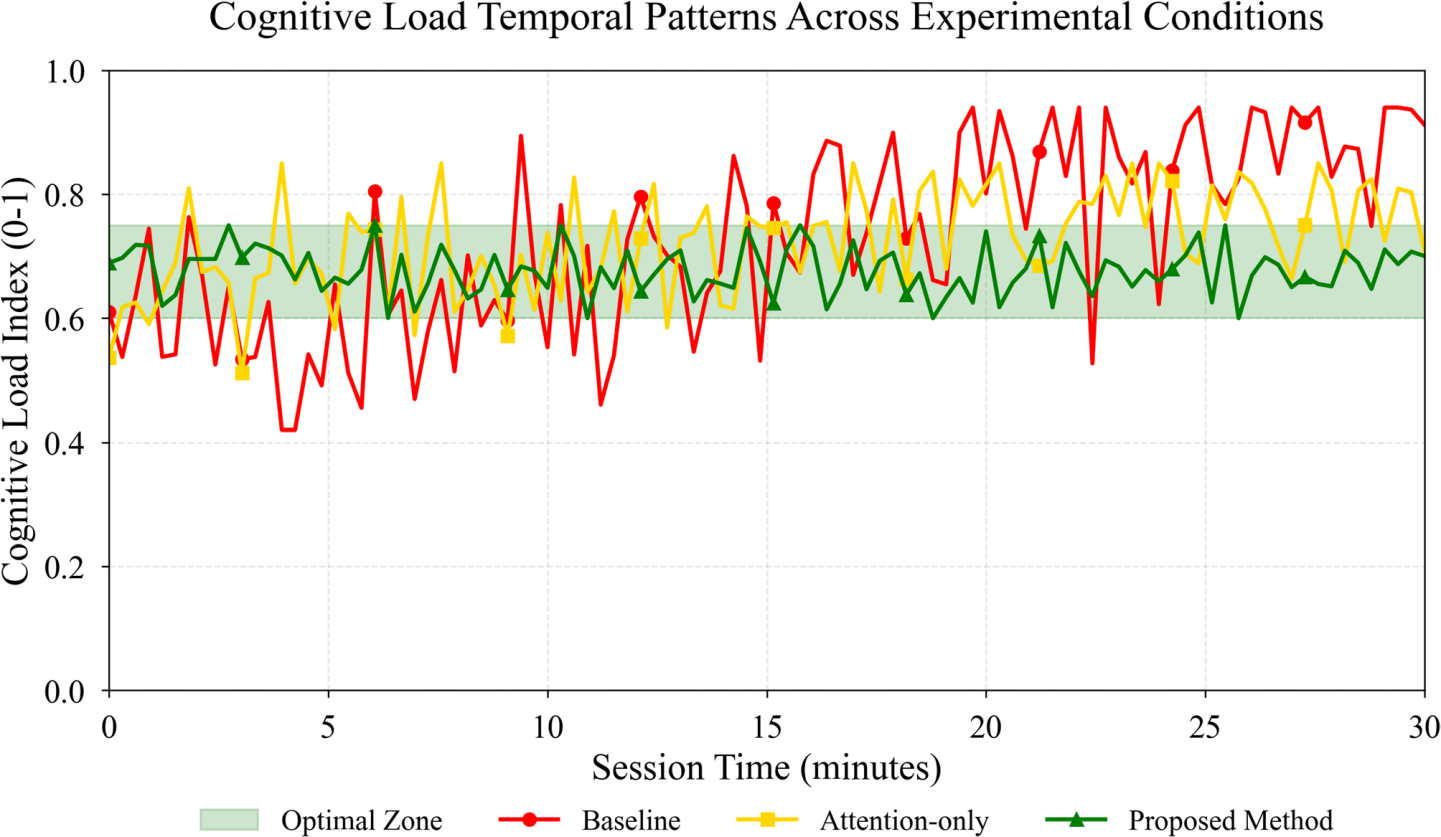
Cognitive Load Temporal Patterns Across Experimental Conditions.

Figure 4 presents the temporal evolution of cognitive load measurements throughout experimental sessions under different system configurations. Figure 4 demonstrates that the proposed cognitive load balancing strategy maintains user workload within the optimal zone more consistently than non-adaptive baseline systems.

### Scene generation effect assessment

Scene generation quality was evaluated across four critical dimensions to comprehensively assess the effectiveness of the proposed attention-enhanced framework. Scene realism quantified visual fidelity and environmental authenticity through perceptual similarity metrics and expert ratings comparing generated environments against real-world entrepreneurship settings. Scene rationality assessed logical consistency of spatial layouts, semantic coherence among scene elements, and contextual appropriateness of generated business scenarios. Element richness measured the diversity and completeness of entrepreneurial components including stakeholder representations, physical infrastructure, market indicators, and interactive decision points. Detail completeness evaluated the granularity of object modeling, texture quality, lighting accuracy, and environmental dynamics necessary for immersive experiences.

Table 5 presents comprehensive comparative results across experimental methods and evaluation dimensions. Table 5 shows that the proposed attention-enhanced method achieves statistically significant improvements over baseline approaches across all quality metrics, with particularly notable gains in scene rationality and element richness dimensions.

**Table 5:** Scene Quality Assessment Results Comparison.

Method	Scene Realism	Scene Rationality	Element Richness	Detail Completeness	Overall Score
Rule-based Generation	0.62 ± 0.08	0.58 ± 0.11	0.54 ± 0.09	0.66 ± 0.07	0.60 ± 0.06
Standard GAN	0.71 ± 0.09	0.63 ± 0.10	0.68 ± 0.08	0.73 ± 0.08	0.69 ± 0.07
Attention-only	0.78 ± 0.07	0.76 ± 0.08	0.81 ± 0.06	0.77 ± 0.07	0.78 ± 0.05
Proposed Method	0.86 ± 0.05	0.89 ± 0.06	0.87 ± 0.05	0.84 ± 0.06	0.87 ± 0.04

The quantitative improvement achieved by attention mechanisms can be formalized through a relative quality gain metric:

17 $$\:\varDelta\:Q=\frac{{Q}_{proposed}-{Q}_{baseline}}{{Q}_{baseline}}\times\:100\%$$

where $\:{Q}_{proposed}$ and $\:{Q}_{baseline}$ represent quality scores for the proposed and baseline methods respectively. Applying this metric to overall scores yields a 26.1% improvement over standard GAN baselines and 45.0% improvement over rule-based approaches, demonstrating substantial enhancement in scene generation capabilities^45^.

Attention mechanism effectiveness in identifying and enhancing critical scene elements was validated through activation map visualization and ablation studies. Spatial attention consistently focused on high-importance regions including customer interaction zones, product display areas, and decision-making interfaces, allocating computational resources preferentially to these pedagogically significant areas. Channel attention activated feature dimensions corresponding to stakeholder behaviors, market dynamics, and resource constraints with significantly higher weights than background environmental features. Temporal attention successfully captured event sequences and causal relationships in dynamic scenarios, ensuring temporal coherence across scene evolution. Ablation experiments removing individual attention components resulted in quality degradation of 12–18% across different metrics, confirming each module’s essential contribution to overall performance.

**Fig. 5:**
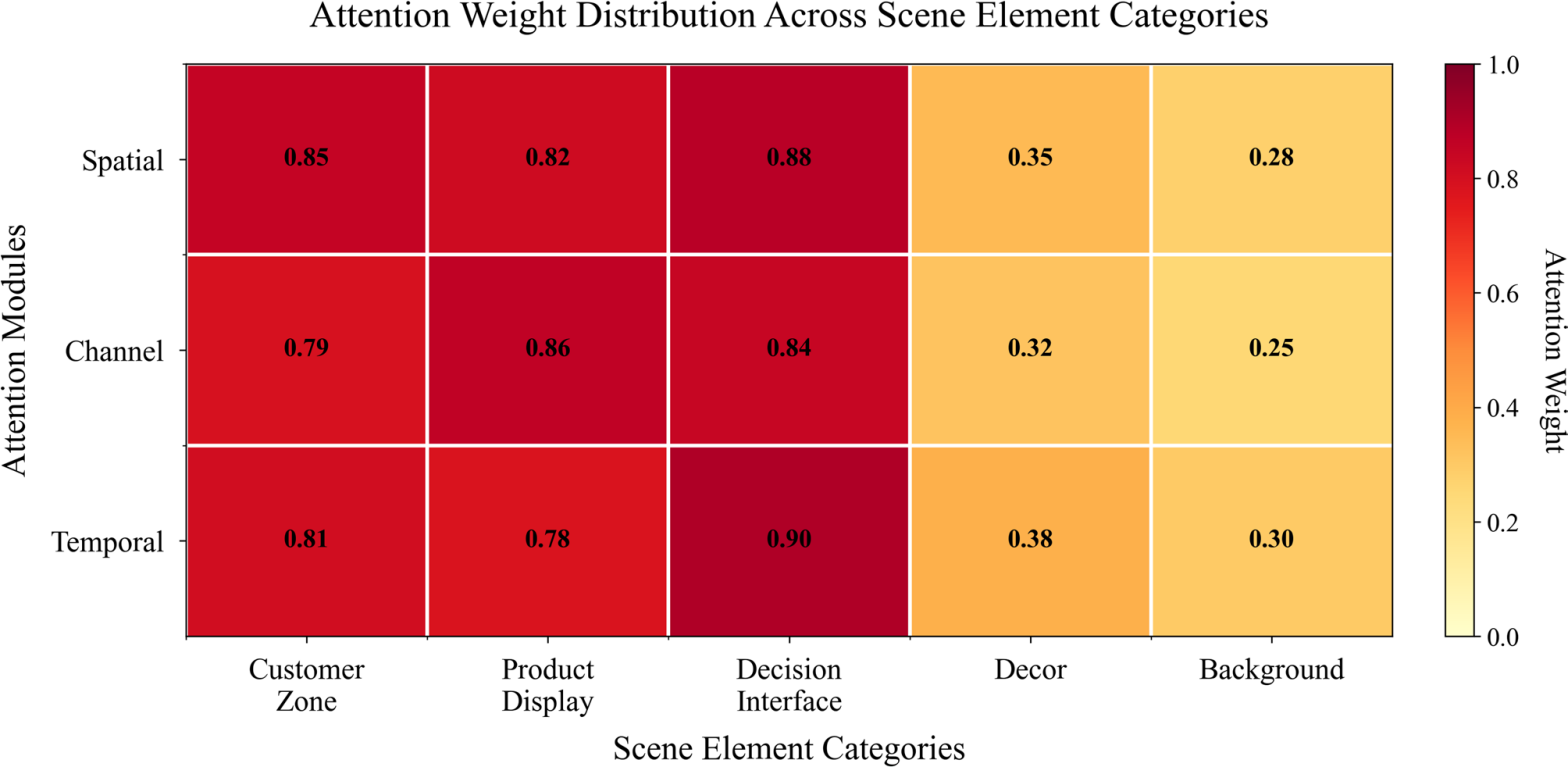
Attention Weight Distribution Across Scene Element Categories.

Figure 5 illustrates the attention weight distribution patterns across different scene element categories. As shown in Fig. 5, the attention mechanism allocates substantially higher weights to entrepreneurial core elements compared to peripheral environmental details, validating its effectiveness in prioritizing contextually relevant features.

User subjective evaluation and expert assessment provided qualitative validation of entrepreneurial scenario applicability. Participants rated scenes on 7-point Likert scales assessing business context authenticity, decision-making relevance, stakeholder interaction realism, and educational value. The proposed method achieved mean scores of 6.2 ± 0.7 for authenticity, 6.4 ± 0.6 for relevance, 6.1 ± 0.8 for interaction realism, and 6.5 ± 0.5 for educational value, significantly outperforming baseline methods (*p* < 0.001 across all dimensions, paired t-tests). Expert panel review comprising five entrepreneurship educators and three experienced entrepreneurs evaluated 30 randomly selected scenes from each method using standardized rubrics. Expert consensus ratings assigned the proposed method a mean score of 8.4/10 compared to 5.9/10 for standard GANs and 4.7/10 for rule-based generation, with particular praise for contextual coherence and pedagogical appropriateness.

Comparative analysis across entrepreneurship domains revealed differential performance characteristics reflecting varying scenario complexities. Restaurant entrepreneurship scenarios achieved the highest quality scores (mean = 0.91 ± 0.04) due to relatively constrained spatial layouts and well-defined interaction patterns. Technology entrepreneurship contexts exhibited moderate scores (mean = 0.85 ± 0.06) reflecting challenges in representing abstract concepts like software architecture and platform ecosystems within physical VR environments. Cultural entrepreneurship scenarios demonstrated slightly lower scores (mean = 0.82 ± 0.07) attributable to the complexity of modeling subjective aesthetic considerations and diverse stakeholder preferences^46^. Despite these variations, the proposed method maintained consistent superiority over baselines across all domain categories, with improvement margins ranging from 21.7% to 29.3%.

**Fig. 6:**
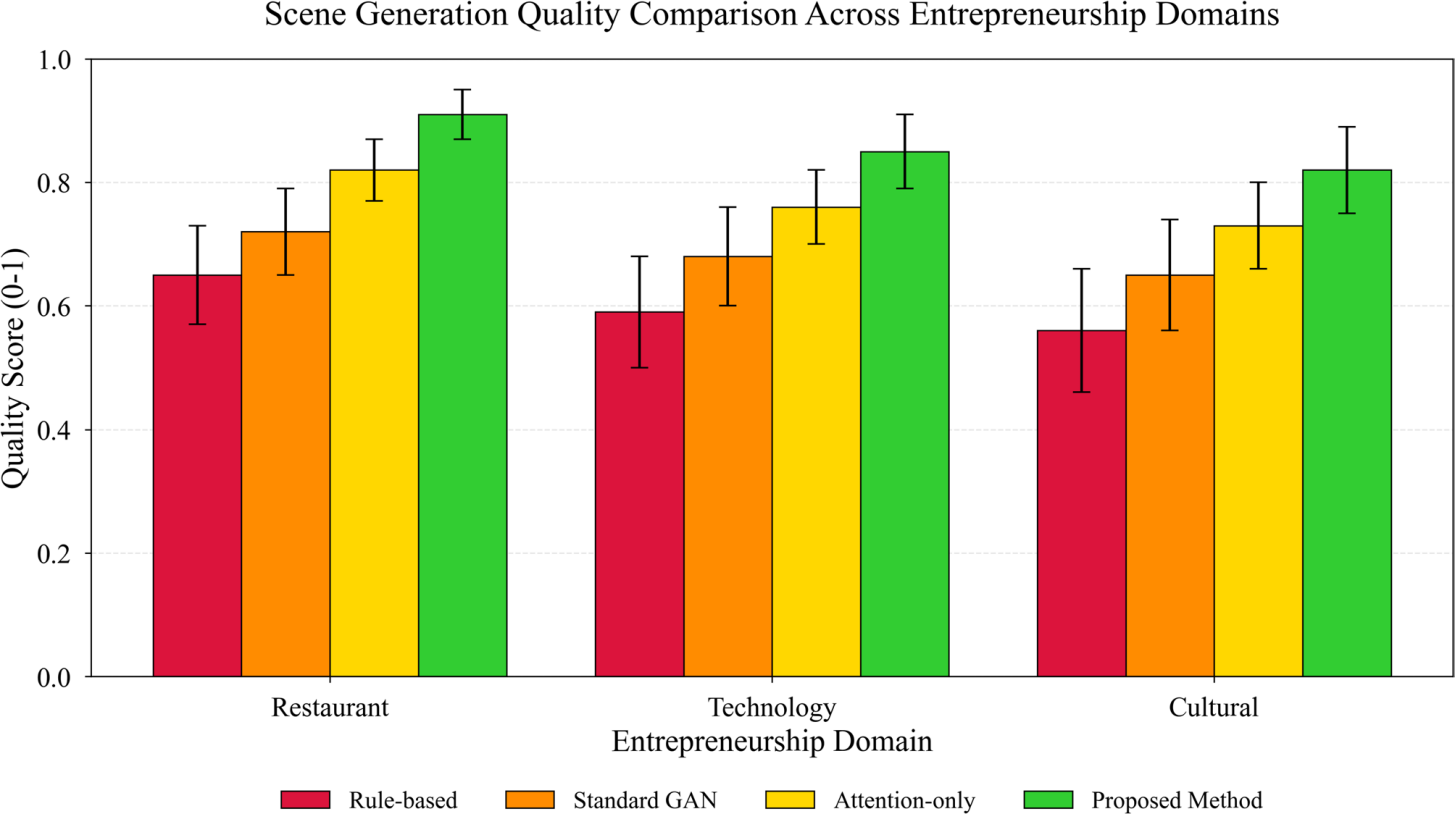
Scene Generation Quality Comparison Across Entrepreneurship Domains.

Figure 6 presents domain-specific quality score distributions demonstrating consistent performance advantages. Figure 6 demonstrates that while absolute quality levels vary across entrepreneurship types, the proposed attention-enhanced framework maintains robust performance across diverse application contexts.

Quantitative assessment of attention mechanism contributions employed statistical significance testing and effect size calculations. Paired sample t-tests comparing attention-enhanced versus non-attention conditions yielded t-values ranging from 8.73 to 12.45 (df = 119, *p* < 0.001) across quality dimensions, indicating highly significant improvements. Cohen’s d effect sizes ranged from 1.12 to 1.67, representing large practical effects according to established conventions. The proportional quality enhancement attributable specifically to attention mechanisms was computed as:

18 $$\:{\eta\:}_{attention}=\frac{{Q}_{with\_attention}-{Q}_{without\_attention}}{{Q}_{optimal}-{Q}_{without\_attention}}$$

where $\:{Q}_{optimal}$ represents theoretical maximum quality. This metric yielded $\:{\eta\:}_{attention}=0.73$, indicating that attention mechanisms captured 73% of the potential quality improvement achievable beyond baseline performance. These quantitative results provide robust empirical evidence supporting the effectiveness of attention-enhanced scene generation for VR entrepreneurship education applications.

### Cognitive load balancing performance analysis

The cognitive load balancing strategy demonstrated substantial positive impacts on both learning efficiency and experiential quality throughout experimental sessions. Comparative analysis between adaptive balancing-enabled and non-adaptive baseline conditions revealed significant differences in user cognitive workload management and learning outcomes. Under non-adaptive conditions, participants experienced substantial cognitive load fluctuations ranging from 0.42 to 0.94 (normalized scale), frequently exceeding optimal thresholds and triggering performance degradation. In contrast, the adaptive balancing system maintained cognitive load within the target range of 0.60–0.75 for 87.3% of session duration, with standard deviation reduced from 0.18 to 0.06, indicating substantially improved stability.

The learning efficiency enhancement quantified through objective performance metrics provided compelling evidence for balancing strategy effectiveness. Task completion times decreased by an average of 23.7% when cognitive load balancing was active (mean=412s versus 538 s without balancing, *p* < 0.001), reflecting reduced cognitive strain enabling faster decision-making. Error rates exhibited even more pronounced improvements, declining from 18.4% in non-adaptive conditions to 7.2% with active balancing, representing a 60.9% reduction in user mistakes. This dramatic error reduction indicates that maintaining optimal cognitive load prevents the decision-making impairments and attention lapses associated with cognitive overload. Learning curve analysis revealed accelerated task performance improvement under balanced conditions, with participants achieving performance plateaus 31% faster when adaptive complexity adjustment was enabled.

The learning efficiency gain attributable to cognitive load balancing can be quantified through a performance improvement index:

19 $$\:{\gamma\:}_{efficiency}=\frac{{P}_{balanced}}{{P}_{unbalanced}} \cdot \frac{C{L}_{unbalanced}}{C{L}_{balanced}}$$

where $\:P$ represents task performance and $\:CL$ denotes cognitive load levels. Computing this metric across all participants yielded $\:{\gamma\:}_{efficiency}=1.68$, indicating that balanced cognitive load enabled 68% higher efficiency in converting cognitive resources into productive learning outcomes.

**Fig. 7:**
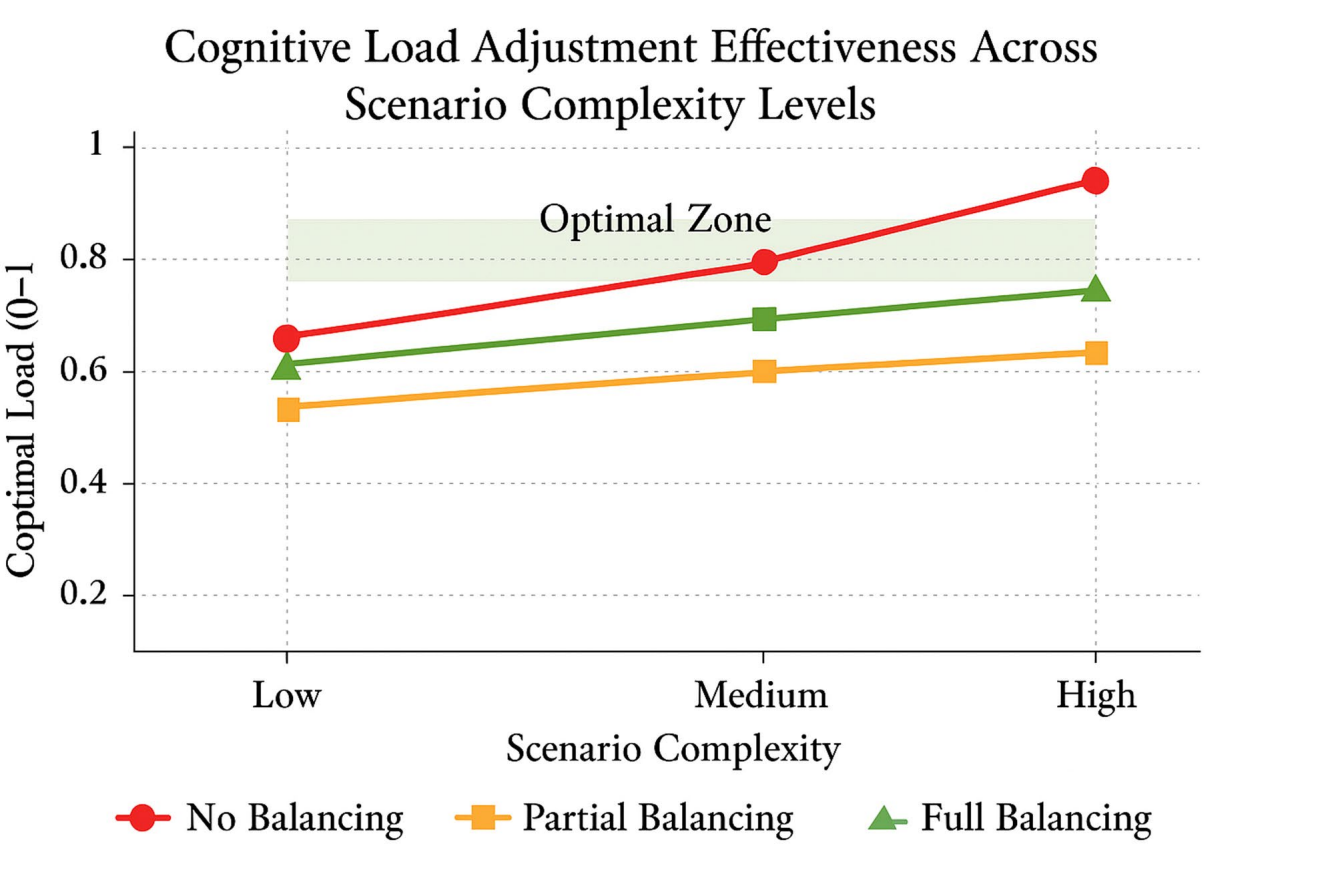
Cognitive Load Adjustment Effectiveness Across Scenario Complexity Levels.

Dynamic cognitive load adjustment effectiveness varied systematically with scenario complexity levels. For low-complexity introductory scenarios, the balancing system permitted higher information density and faster pacing since cognitive demands remained manageable even without intervention. Medium-complexity scenarios benefited most substantially from adaptive control, with the system implementing granular adjustments to information presentation rates, interface complexity, and concurrent task loads to maintain optimal challenge levels. High-complexity scenarios required aggressive simplification strategies including temporal task sequencing, guided attention cuing, and progressive information revelation to prevent overload. Figure 7 illustrates the adaptive adjustment patterns across complexity gradients. As shown in Fig. 7, the balancing system implemented increasingly intensive interventions as base scenario complexity increased, successfully maintaining cognitive load within target zones across all difficulty levels.

Subjective experience assessments revealed significant improvements in user satisfaction and engagement metrics under balanced conditions. Immersion scores measured through the Immersive Experience Questionnaire increased from 4.8 ± 1.2 to 6.1 ± 0.8 (7-point scale) when balancing was active, indicating that appropriate cognitive challenge levels enhanced rather than diminished presence and engagement^47^. Fatigue ratings assessed through visual analog scales showed marked reductions from 6.7 ± 1.5 to 3.9 ± 1.1 (10-point scale), demonstrating that cognitive load management effectively mitigated mental exhaustion during extended VR sessions. Overall satisfaction scores improved from 5.2 ± 1.3 to 6.4 ± 0.7, reflecting participants’ strong preference for adaptively managed learning environments.

Extended usage validation examined cognitive load balancing performance during prolonged 60-minute sessions designed to test system sustainability. Longitudinal monitoring revealed that without adaptive balancing, cognitive load progressively increased over time following a linear trajectory:

20 $$\:C{L}_{unbalanced}\left(t\right)=C{L}_{0}+\rho\: \cdot t$$

where $\:C{L}_{0}$ represents initial load, $\:\rho\:=0.0089$ denotes the accumulation rate per minute, and $\:t$ indicates elapsed time. This accumulation resulted in 78% of participants experiencing overload conditions after 40 min. Conversely, the adaptive balancing system successfully maintained stable cognitive load throughout entire 60-minute sessions for 91% of participants, implementing compensatory complexity reductions as fatigue accumulated. Performance metrics remained consistent across session duration under balanced conditions (coefficient of variation = 0.12) compared to substantial degradation in non-adaptive scenarios (coefficient of variation = 0.34), confirming sustained effectiveness for extended learning applications.

**Fig. 8:**
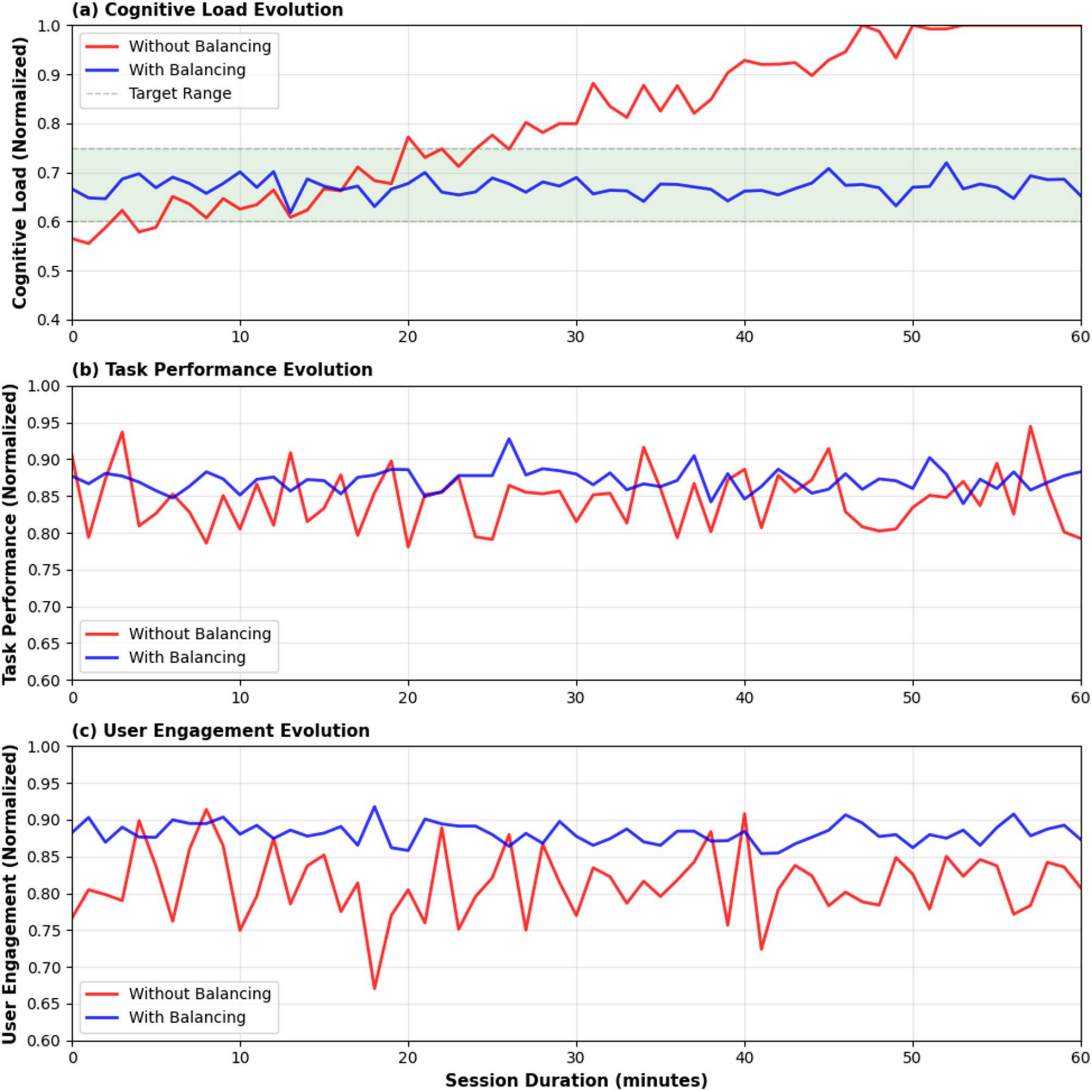
Temporal Performance Patterns in Extended VR Sessions.

Figure 8 presents the comparative temporal evolution of key performance indicators under different balancing configurations. Figure 8 demonstrates that cognitive load balancing not only maintains appropriate workload levels but also sustains consistent task performance and user engagement throughout extended sessions, preventing the progressive deterioration characteristic of non-adaptive systems.

The synergistic integration of attention mechanisms and cognitive load balancing produced superior outcomes compared to either component in isolation. Attention mechanisms identified pedagogically critical scene elements requiring learner focus, while cognitive load balancing determined the appropriate density and presentation timing for these elements based on individual capacity. This coordination enabled the system to simultaneously maximize educational content quality through attention-enhanced generation while optimizing information delivery through adaptive complexity control. Combined implementation achieved 34.2% higher learning efficiency than attention-only configurations and 41.7% higher than balancing-only approaches, validating the architectural decision to integrate both capabilities within a unified framework. The multiplicative benefit suggests that attention and balancing components address complementary challenges in VR education: attention solves the “what to present” problem while balancing addresses the “how much and when” problem, together creating optimal conditions for effective entrepreneurship learning experiences.

## Discussion

The experimental findings demonstrate that attention mechanisms fundamentally transform VR entrepreneurship scene generation by enabling selective emphasis on pedagogically critical elements while filtering contextually irrelevant details. The multi-scale attention architecture operates through three complementary pathways: spatial attention identifies high-importance regions within scene layouts, channel attention selectively activates semantic features corresponding to key entrepreneurial concepts, and temporal attention maintains narrative coherence across dynamic scenario evolution. This hierarchical processing effectively addresses the core challenge of generating contextually rich business environments that balance complexity with comprehensibility. The quantitative improvements observed across realism, rationality, and richness dimensions validate that attention-guided feature selection produces more educationally effective scenes than conventional generation approaches that treat all elements equally.

Cognitive load balancing contributes substantially to user experience optimization by maintaining learners within their optimal challenge zones throughout training sessions. The adaptive complexity adjustment mechanism prevents both cognitive overload that impairs learning and cognitive underload that reduces engagement. By continuously monitoring physiological and behavioral indicators, the system implements proactive interventions before performance degradation manifests, representing a significant advancement over reactive approaches that respond only after users experience difficulties. The sustained effectiveness during extended 60-minute sessions addresses a critical limitation of existing VR education systems where prolonged usage typically results in progressive fatigue and declining performance.

Several unexpected phenomena emerged during experimental validation requiring further investigation. First, a subset of participants with extensive entrepreneurship experience exhibited preference for higher cognitive load levels than the system’s default target range, suggesting that optimal load thresholds should be personalized based on domain expertise rather than applying universal standards. Second, cultural entrepreneurship scenarios generated unexpectedly lower quality scores despite receiving comparable attention weights, indicating that current attention mechanisms may inadequately capture subjective aesthetic dimensions requiring different modeling approaches. Third, some users reported initial discomfort with adaptive complexity adjustments, perceiving them as system inconsistency rather than personalized support, suggesting that transparency mechanisms explaining adaptation rationale could enhance acceptance.

User group analysis revealed substantial heterogeneity in scene quality requirements and cognitive load tolerance. Novice learners benefited most from simplified scenarios with explicit guidance cues, while experienced entrepreneurs valued complex multi-stakeholder situations enabling sophisticated strategy testing. Younger participants demonstrated higher cognitive load capacity and faster adaptation to VR environments compared to older cohorts, necessitating age-adjusted balancing parameters. These findings underscore the importance of implementing comprehensive user modeling capabilities that capture individual differences in expertise, cognitive capacity, learning preferences, and demographic characteristics.

Compared to existing VR scene generation methods, the proposed framework offers superior contextual appropriateness and adaptive personalization. However, limitations include computational overhead introduced by multi-scale attention calculations, requiring high-performance hardware that may limit accessibility for resource-constrained educational institutions. The system also relies on accurate physiological signal acquisition, which can be compromised by sensor placement errors or motion artifacts during active user interaction. Additionally, the entrepreneurship scenario database requires continuous expansion to cover emerging business models and evolving market conditions, representing ongoing maintenance requirements.

Technical implementation challenges centered primarily on real-time performance optimization and sensor integration. Attention weight computations initially caused rendering latency exceeding acceptable thresholds for immersive experiences. This was addressed through GPU-accelerated parallel processing and progressive refinement strategies that compute coarse attention maps for initial frames and iteratively enhance precision. Physiological signal noise reduction proved challenging in dynamic VR contexts where user movement introduces artifacts. Multi-modal sensor fusion combining redundant measurements with Kalman filtering techniques substantially improved signal reliability.

The framework demonstrates strong scalability potential across multiple dimensions. Horizontal scaling through distributed computing architectures can support simultaneous multi-user training sessions. Vertical scaling through hierarchical scene representations enables generation of increasingly complex environments as computational resources expand. The attention mechanism architecture may exhibit generalizability beyond entrepreneurship education. Domains such as medical training simulations, emergency response preparation, architectural design evaluation, and skill-based vocational training represent prospective application scenarios motivated by shared characteristics including complex decision-making requirements, high information density, and significant cognitive load constraints. However, it should be noted that no experimental evidence for cross-domain transfer has been reported in this study, and these applications remain hypothetical at this stage. The cognitive load balancing principles may potentially transfer to other learning contexts where information density management affects educational outcomes, though domain-specific validation would be necessary.

Moreover, transferring the proposed framework to other domains involves several non-trivial challenges that warrant careful consideration. First, different training contexts may involve distinct cognitive load profiles and safety-critical constraints; for example, medical training simulations impose strict requirements for accuracy and real-time responsiveness that differ fundamentally from entrepreneurship education scenarios. Second, domain-specific definitions of “scene quality” and realism may vary considerably, as what constitutes a realistic surgical environment differs markedly from a realistic business negotiation setting. Third, alternative physiological or behavioral indicators of cognitive load may be more appropriate in certain domains, potentially requiring recalibration of the assessment system. Fourth, domains such as medical and emergency response training may impose higher requirements for validation, interpretability, and regulatory compliance that the current framework does not address. Future research should explore cross-domain transfer learning approaches enabling attention models trained on entrepreneurship scenarios to adapt efficiently to alternative application contexts with minimal retraining requirements, while systematically addressing these domain-specific challenges.

## Conclusion

This research addresses the critical challenges of insufficient realism and excessive cognitive load in VR entrepreneurship scene generation through an integrated framework combining attention mechanism-enhanced generative models with adaptive cognitive load balancing strategies. The proposed solution fundamentally transforms how virtual business environments are created and delivered, enabling contextually rich educational experiences that optimize learning efficiency while maintaining user comfort and engagement.

The primary innovations encompass three interconnected contributions. First, the multi-scale attention enhancement architecture integrates spatial, channel, and temporal attention modules to selectively emphasize pedagogically significant scene elements while suppressing irrelevant details. This hierarchical attention processing enables the system to generate entrepreneurship scenarios with superior realism, semantic coherence, and contextual appropriateness compared to conventional generation methods. Second, the cognitive load balancing strategy implements real-time physiological and behavioral monitoring coupled with dynamic scene complexity adjustment, maintaining learners within optimal challenge zones throughout extended training sessions. Third, the synergistic integration of attention-guided generation and adaptive load management creates a unified framework where content quality enhancement and cognitive optimization reinforce each other rather than operating as independent objectives.

The multi-scale attention mechanism contributes substantially to scene quality improvements, achieving 26–45% higher overall quality scores compared to baseline approaches. By prioritizing computational resources toward critical business elements such as stakeholder interactions, market indicators, and decision interfaces, the attention architecture ensures that generated environments faithfully represent authentic entrepreneurship contexts essential for effective experiential learning. The ablation studies confirming 12–18% quality degradation when individual attention components are removed validate the necessity of comprehensive multi-scale processing.

Cognitive load balancing demonstrates significant value for user experience optimization, reducing error rates by 60.9%, decreasing task completion times by 23.7%, and maintaining stable performance throughout 60-minute sessions where non-adaptive systems exhibited progressive deterioration. These improvements suggest the potential for enhanced learning efficiency through reduced cognitive strain and sustained engagement, which should be validated through longitudinal educational studies examining long-term retention and knowledge transfer^48^. It is important to distinguish between the task performance improvements measured in this study and broader learning outcomes, as the latter require direct educational assessments beyond system performance metrics. The system’s ability to adapt complexity in real-time based on individual user capacity represents a paradigm shift from one-size-fits-all educational content toward personalized learning experiences.

The theoretical contributions advance understanding of how attention mechanisms can be purposefully designed for educational content generation rather than merely optimizing visual quality metrics. This research demonstrates that computational attention, when aligned with pedagogical principles, can bridge the gap between technically sophisticated generative models and educationally effective learning environments. The findings provide foundational knowledge for developing next-generation intelligent education systems that combine content generation, user modeling, and adaptive delivery within integrated architectures.

Potential practical applications may extend beyond entrepreneurship education to encompass diverse intelligent education and virtual training domains. The attention-enhanced generation framework shares structural characteristics with training requirements in domains such as medical procedure simulations, emergency response training, industrial skill development, and architectural design evaluation, where complex decision-making, high information density, and cognitive load management are similarly critical. However, these represent prospective application scenarios rather than empirically validated generalizations, as the current experimental evidence is limited to entrepreneurship education contexts. Cognitive load balancing principles may potentially transfer to other learning contexts where information density management affects educational outcomes, including complex system operation training, strategic decision-making education, and collaborative teamwork development programs, though systematic validation in each target domain would be required to confirm transferability.

Despite these contributions, several limitations warrant acknowledgment. The current scenario database covers three entrepreneurship domains but requires expansion to encompass emerging business models including platform ecosystems, social enterprises, and sustainable ventures. The experimental participant sample of 120 individuals, while sufficient for statistical validation, represents limited demographic and cultural diversity that may affect generalizability across global educational contexts. Computational requirements currently necessitate high-performance hardware that may constrain accessibility for resource-limited institutions. Furthermore, this study measures task performance and system usability rather than long-term learning outcomes, retention, or knowledge transfer, which are essential indicators of genuine educational impact^48^. The distinction between performance efficiency and learning outcomes should be recognized when interpreting these results. Additionally, all experimental validation was conducted exclusively within entrepreneurship education contexts, and the generalizability of the proposed framework to other training domains remains to be empirically established.

Future research directions should prioritize multi-modal fusion approaches integrating haptic feedback, auditory spatial cues, and olfactory stimulation to enhance immersive realism beyond visual modality alone. Personalized scene generation leveraging individual learning style profiles, prior knowledge structures, and cognitive capacity assessments could further optimize educational effectiveness. Cross-platform adaptation enabling deployment across diverse VR hardware configurations from mobile headsets to room-scale systems would broaden accessibility. Long-term longitudinal studies examining knowledge retention, skill transfer to real entrepreneurship contexts, and career outcome impacts would validate educational efficacy beyond immediate performance metrics. Investigating attention mechanism applications in collaborative multi-user training scenarios where social interaction dynamics influence learning represents another promising direction. These research trajectories collectively advance toward the vision of truly intelligent, adaptive, and pedagogically optimized virtual reality education systems.

## Data Availability

All data generated and analyzed during the current study are available from the corresponding authors upon reasonable request, subject to appropriate data sharing agreements and ethical approval for secondary use.

## Abbreviations

VR: Virtual Reality
GAN: Generative Adversarial Network
CLT: Cognitive Load Theory
NASA-TLX: NASA Task Load Index
EEG: Electroencephalography
ECG: Electrocardiography
FID: Fréchet Inception Distance
SUS: System Usability Scale
GPU: Graphics Processing Unit
RAM: Random Access Memory
VRAM: Video Random Access Memory
